# Dexamethasone for treatment of severe COVID-19, a surprise?

**DOI:** 10.1186/s43057-020-00032-1

**Published:** 2020-09-24

**Authors:** Hany Hasan Elsayed

**Affiliations:** Thoracic Surgery Department (ARDS Taskforce), Faculty of Medicine, Ain Shams University, Abbasia Square, Cairo, Egypt

**Keywords:** COVID-19, ARDS, Dexamethasone

The inclination that we lack all the knowledge needed to treat COVID-19 related ARDS may be dangerous in depriving COVID-19 patients from evidence-based medication.

The Berlin criteria defined ARDS as an acute syndrome of hypoxia (P/F ratio less than 300) with bilateral lung opacities on imaging not fully explained by a cardiogenic cause or fluid overload [1]. Currently, the majority of patients with COVID-19 respiratory failure exhibit a similar gas exchange, respiratory system mechanics, and response to prone ventilation as prior large cohorts of patients with ARDS.

Although most severe COVID-19 patients will fulfil the classic definition of ARDS, Gattinoni et al. [2] suggested that a subset of COVID-19 pneumonia patients have preserved lung compliance and present with “silent hypoxaemia”. They suggested the presence of an L-phenotype in an interesting article that stimulates thought and suggests a framework for how to manage COVID-19 patients. The problems were the small number of patients and that “compliance” does not fit in the Berlin criteria, so we still need to categorise these patients as having ARDS.

In fact, the Surviving Sepsis Campaign panel recently recommended that “mechanically ventilated patients with COVID-19 related ARDS should be managed similarly to other patients with acute respiratory failure in the ICU” [3].

ARDS has always been a construct in the minds of clinicians and researchers. It exists not because it is perfect, but because it has utility. It has utility for clinicians as it gives us a frame of reference for categorising patients, providing appropriate therapies, and prognosticating. It has utility for research as it allows otherwise heterogeneous patient groups to be studied in adequately powered clinical trials and provides a touchstone for new concepts and discoveries.

The role of steroids in reducing mortality and ventilation days in patients with ARDS is well established [4, 5]. In fact, Villar and his colleagues have published the largest meta-analysis of using dexamethasone treatment for the acute respiratory distress syndrome few months back and this has shown a mortality benefit [6].

The RECOVERY trial (www.recoverytrial.net) is the largest existing randomised controlled trial to find the best treatment for COVID-19 patients. It is UK based and expected to recruit 11,500 patients to six different treatment arms in addition to standard treatment in each hospital: no additional treatment vs lopinavir-ritonavir vs low-dose corticosteroids vs hydroxychloroquine vs azithromycin. In a factorial design, eligible patients are allocated simultaneously to no additional treatment vs convalescent plasma.

A report announced from the main trial investigator on 16 June 2020 stated that a total of 2104 patients were randomised to receive dexamethasone 6 mg once per day (either by mouth or by intravenous injection) for 10 days and were compared with 4321 patients randomised to usual care alone. Among the patients who received usual care alone, 28-day mortality was highest in those who required ventilation (41%), intermediate in those patients who required oxygen only (25%), and lowest among those who did not require any respiratory intervention (13%). Dexamethasone reduced deaths by one third in ventilated patients (rate ratio 0.65 [95% confidence interval 0.48 to 0.88]; *p* = 0.0003) and by one fifth in other patients receiving oxygen only (0.80 [0.67 to 0.96]; *p* = 0.0021). There was no benefit among those patients who did not require respiratory support (1.22 [0.86 to 1.75]; *p* = 0.14). Based on these results, 1 death would be prevented by treatment of around 8 ventilated patients.

This is concomitant with all the previous evidence of the role of steroids in ARDS patients. In fact, 1772 patients with severe COVID-19 ARDS requiring mechanical ventilation in the RECOVERY trial did not receive steroids. If we have solid evidence that dexamethasone is beneficial for patients with established ARDS due to a variety of causes and we believe that patients with severe COVID-19 disease develop ARDS, how can we deprive five out of six arms of treatment in the RECOVERY trial who will develop COVID-19 related ARDS from steroids (or namely dexamethasone)?

I believe that most intensivists around the world were using dexamethasone for their patients with severe COVID-19 developing ARDS before the RECOVERY trial results were released based on the evidence they had on how to manage this unique entity from a variety of causes. The question will remain: are we in a breakthrough of a new medication giving hope to millions around the world awaiting an efficient medication for COVID-19 or are we solidifying the evidence we already know about what is effective for an ARDS patient regardless of its cause?

## Data Availability

All data and material are available on request.

## Abbreviations

ARDS: Adult respiratory distress syndrome
ICU: Intensive care unit
